# Ectopic lymphoid follicles: inducible centres for generating antigen‐specific immune responses within tissues

**DOI:** 10.1111/imm.12554

**Published:** 2015-12-10

**Authors:** Gareth W. Jones, Simon A. Jones

**Affiliations:** ^1^ Division of Infection and ImmunityThe School of MedicineCardiff UniversityCardiffUK

**Keywords:** arthritis, autoimmunity, cancer, infection, lymphoid neogenesis

## Abstract

Lymphoid neogenesis is traditionally viewed as a pre‐programmed process that promotes the formation of lymphoid organs during development. Here, the spatial organization of T and B cells in lymph nodes and spleen into discrete structures regulates antigen‐specific responses and adaptive immunity following immune challenge. However, lymphoid neogenesis is also triggered by chronic or persistent inflammation. Here, ectopic (or tertiary) lymphoid organs frequently develop in inflamed tissues as a response to infection, auto‐immunity, transplantation, cancer or environmental irritants. Although these structures affect local immune responses, the contribution of these lymphoid aggregates to the underlining pathology are highly context dependent and can elicit either protective or deleterious outcomes. Here we review the cellular and molecular mechanisms responsible for ectopic lymphoid neogenesis and consider the relevance of these structures in human disease.

## Secondary and ectopic lymphoid organs

Secondary lymphoid organs (SLOs) are responsible for immune homeostasis and the development of adaptive immune responses to invading pathogens.^1^ Here, the accumulation of foreign antigens within the highly organized cellular architecture of SLOs facilitates antigen presentation to T and B cells and the establishment of adaptive immunity. Encapsulated SLOs form at predetermined locations during embryonic development and include the spleen and lymph nodes. Lymph nodes are strategically located throughout the body to monitor self and non‐self antigens displayed by antigen‐presenting cells as they are trafficking from peripheral organs and tissue. The spleen is also important for protection against pathogens carried in the blood. SLOs also include the non‐encapsulated mucosal‐associated lymphoid tissues that feature at barrier surfaces and include Peyer's patches, tonsils, nasal‐associated lymphoid tissue and bronchus‐associated lymphoid tissue (BALT). These latter types of SLOs are found in the sub‐mucosal epithelium and are responsible for preserving tissue integrity at barrier surfaces by ensuring the maintenance of immune tolerance against protective commensal microbiota and host responses to pathogenic insult.^2^

To generate fast and efficacious anti‐pathogen responses, lymphoid organs have evolved to maximize encounters between lymphocytes and antigen‐loaded antigen‐presenting cells. Consequently, lymphoid organs share a cellular organization that includes a germinal centre comprising antibody secreting and proliferating B cells together with follicular dendritic cells (DCs); a T‐cell zone including naive cells recruited from the blood; high endothelial venules (HEV) for lymphocyte extravasation; and a network of stromal cells that provide chemokines and extracellular matrix for cellular migration and structural integrity.^1^, ^3^

Inflammation is the consequence of our immunological response to infection, autoimmunity, cancer, injury and allograft transplantation.^4^ Appropriate control of inflammation ensures competent host defence and is governed by cellular communication between non‐haematopoietic stromal cells, tissue‐resident leukocytes and infiltrating immune cells.^4^, ^5^, ^6^, ^7^ However, inappropriate control, for example during autoimmunity, results in sustained immune responses causing chronic inflammation. Without therapeutic intervention, over time this inflammation drives clinical symptoms that culminate in tissue destruction and loss of function.^8^ Leukocyte infiltration is classically viewed as a random, diffuse accumulation of cells within affected tissues. However, there is emerging appreciation that during chronic inflammation, infiltrating immune cells can form highly organized aggregates of lymphoid cells that resemble SLOs. These ectopic lymphoid follicles (ELFs), also known as tertiary lymphoid structures, can propagate local antigen‐specific responses within tissues.^9^, ^10^ Occasionally, these ELFs are named according to their site of development (e.g. inducible bronchus‐associated lymphoid tissue; iBALT). Whereas SLOs develop during ontogeny, ELFs are ‘induced’ in response to an inflammatory insult within target tissues. This is particularly the case where there is a perceived need for sustained leukocyte extravasation due to the failure to clear antigen. Such responses often occur at sites of infection, autoimmunity, cancer, allograft rejection or continued insult from environmental irritants. Consequently, ELFs are ‘transient’ structures, and often resolve upon successful antigen clearance. So what controls the development of these structures in inflamed tissues? While the molecular signatures associated with ELFs resemble those involved in SLO formation, the development or maintenance of ELFs in these sites is significantly influenced by the nature of the local tissue microenvironment. For example, various novel immune subsets have recently been identified as inducers of ELF development that are distinct from the lymphoid tissue inducer (LTi) cells involved in secondary lymphoid organogenesis. The discovery of these subsets now provides new opportunities and therapeutic strategies for targeting ELF‐driven pathologies with biological drugs.

Here we review the cellular and molecular regulators that govern ELF development, their functional importance in disease and how ELFs impact the application of biological drug interventions in chronic disease and cancers.

## Cellular initiators of ectopic lymphoneogenesis

Given that only a proportion of patients suffering any particular inflammatory condition will develop ELFs – for example, approximately 40% of patients with rheumatoid arthritis develop synovial ELFs^11^ – ectopic lymphoid neogenesis must be controlled by a specific set of inflammatory signals. Likewise, as some tissues and tumours are more permissive to ELF development than others, the tissue microenvironment must contribute defined signals that are conducive to lymphoid neogenesis. In this regard, the development of ELFs mimics many of the mechanisms underpinning the organogenesis of SLOs (for a comprehensive review of SLO development see refs 1, 12). Here, initiation of SLO development centres on an interaction at the lymph node anlagen between haematopoietic derived CD4^+^ CD45^+^ CD3^−^ LTi cells and lymphoid tissue organizer (LTo) cells of mesenchymal origin. Here, LTi cells accumulate in response to the local expression of CXCL13, interleukin‐7 (IL‐7) and receptor activator of nuclear factor‐*κ*B ligand (RANKL; also called TNFSF11), owing to the cell surface expression of CXCR5 and the IL‐7 receptor (also called CD127). In response to IL‐7 and RANKL, LTi cells secrete lymphotoxin (LT) *α*_1_ *β*_2_, which engages the LT*β* receptor (LT*β*R) expressed on LTo cells. In turn, LTo cells release the homeostatic chemokines CXCL13, CCL19 and CCL21 in order to recruit haematopoietic cells and up‐regulate the expression of the adhesion molecules vascular cell adhesion molecule 1, intercellular adhesion molecule 1 and mucosal addressin cell adhesion molecule‐1 to ensure lymphocyte retention during SLO development.^13^, ^14^ LTo cells also secrete vascular growth factor‐C, fibroblast growth factor‐2 and hepatocyte growth factor, which promote the development of the lymphatic vasculature and HEVs.^12^, ^14^ Stromal LTo cells also differentiate into stromal cell lineages including follicular DCs, fibroblastic reticular cells and marginal reticular cells, which populate lymph nodes and contribute to SLO function.^12^, ^15^, ^16^

**Figure 1:**
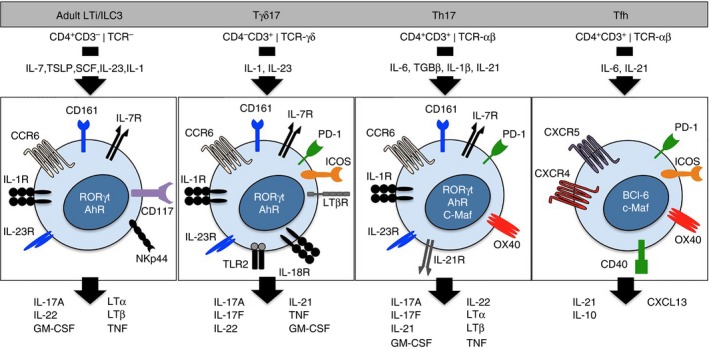
Novel immune cell subsets implicated in the regulation of ectopic lymphoid follicles (ELFs). Novel innate and adaptive immune cell subsets have recently been implicated in ELF regulation. These include the adult lymphoid tissue inducer (LTi) ‐like or innate lymphoid 3 cells, interleukin‐17 (IL‐17) ‐producing *γδ*T (T*γδ*17) cells, T helper type 17 (Th17) cells and follicular T helper (Tfh) cells. Here we highlight the similarities in their phenotype including the cytokines involved in their development, proliferation and effector function, the receptors expressed on the cell surface and the effector cytokines produced by these cells. Similarities in the effector characteristics of these cells may account for their common ability to regulate ELF development or activity. TSLP, thymic stromal lymphopoietin; SCF, stem cell factor; GM‐CSF, granulocyte–macrophage colony‐stimulating factor; TNF, tumour necrosis factor; PD‐1, programmed cell death‐1; ICOS, inducible T‐cell co‐stimulator.

There is increasing evidence that immune cells recruited to inflammatory lesions initiate ELF development (Fig. 1). For example, IL‐17‐secreting CD4 T helper (Th17) cells have been extensively linked with ELF development in experimental models of chronic inflammation.^17^ Here, the development of iBALT as a consequence of pulmonary inflammation was dependent on the Th17 signature cytokine IL‐17, which caused an LT*α*‐independent induction of the lymphoid chemokine CXCL13.^18^ This demonstrates the ability of this effector T helper cell to initiate ELF development. Notably, the adoptive transfer of *in vitro* generated Th17 cells into mice is also sufficient to drive ELF development in a model of multiple sclerosis.^19^ The expression of the cell surface glycoprotein podoplanin (also called gp38) by Th17 cells was required for the development of these lymphoid follicles in the central nervous system. Indeed, mice deficient in podoplanin, or its receptor CLEC‐2, display a defect in the development and maintenance of lymph nodes.^13^, ^19^, ^20^ Our recent study of synovial ELF development in IL‐27R‐deficient mice with inflammatory arthritis identified podoplanin‐expressing T cells within synovial lymphoid aggregates and described IL‐27 as a negative regulator of podoplanin‐expressing Th17 cells.^21^

**Figure 2:**
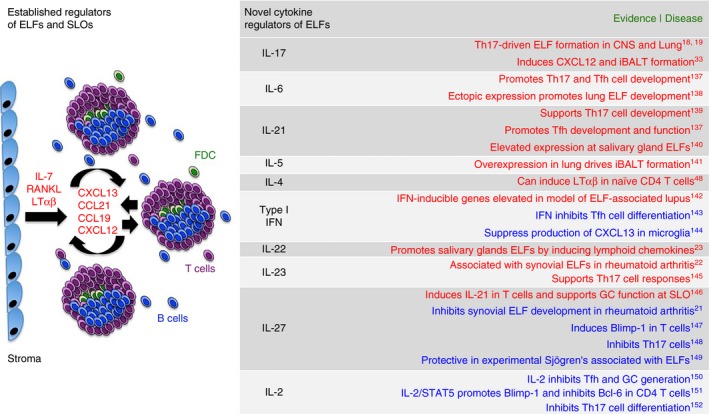
Novel cytokine regulators of ectopic lymphoid follicle (ELF) development and function. The formation of ELFs at sites of chronic inflammation mirrors the pre‐programmed development of conventional secondary lymphoid organs (SLOs). During secondary lymphoid organogenesis, the cytokines interleukin‐7 (IL‐7), receptor activator of nuclear factor‐*κ*B ligand (RANKL) and lymphotoxin (LT) *αβ* initiate the chemokine‐directed positive feedback loop that drives B‐cell, T‐cell and follicular dendritic cell (DC) recruitment during lymphoid neogenesis. Recent studies have implicated novel T helper cell subsets as initiators of ELF formation. Given the role that cytokines play in the regulation of T helper cell differentiation and effector function, a number of cytokines have now been linked with the control of ELFs. For example, cytokines involved in the regulation of T helper type 17 (Th17) cell responses (IL‐6, IL‐21, IL‐23, IL‐27, IL‐2, IL‐22, IL‐17)^23^, ^137^, ^139^, ^145^, ^148^, ^152^ and follicular T helper (Tfh) cell responses [IL‐6, IL‐21, Type I interferons (IFNs), IL‐27, IL‐2]^137^, ^139^, ^146^, ^147^, ^150^ are emerging as regulators of lymphoid neogenesis. Here we highlight cytokines that may positively (red) and negatively (blue) control ELFs based on their ability to regulate effector T‐cell populations involved in ELF development or function. These cytokines, as well as their downstream signalling pathways and transcription factors, have the potential to serve as therapeutic targets in clinical conditions where ELFs feature.

Recently, other cytokines linked with the IL‐17/Th17 cell axis have also been associated with control of lymphoid neogenesis (Fig. 2). For example, IL‐23 is linked with ectopic lymphoid neogenesis in rheumatoid arthritis.^22^ Through control of lymphoid chemokine production in epithelial and fibroblastic stromal cells, IL‐22 also drives lymphoid neogenesis in mice following salivary gland cannulation with adenovirus.^23^ Podoplanin and IL‐17 have also been linked with ectopic lymphoneogenesis in human diseases.^21^, ^24^, ^25^

It has recently emerged that Th17‐type responses are not solely restricted to conventional T helper cells. Adult LTi cells, a group‐3 innate lymphoid cell subset, bear many of the features of Th17 cells, which suggests an ancestral link between these cell types.^26^, ^27^ Both cells express the transcriptional regulator retinoic acid receptor‐related orphan receptor *γ*; are responsive to IL‐23 and aryl hydrocarbon receptor ligands; and can produce IL‐17, IL‐22 and granulocyte–macrophage colony‐stimulating factor.^28^, ^29^, ^30^ Like Th17 and fetal LTi cells, innate lymphoid cells have been linked with lymphoid organogenesis. The adoptive transfer of adult CD4^+^ CD3^−^ LTi cells into *Cxcr5* ^*−/−*^ mice induces the development of intestinal lymphoid tissues.^31^ Similarly, the increased availability of IL‐7 in transgenic mice has been associated with the LTi cell‐dependent development of additional Peyer's patches, caecal patches and *de novo* formation of ectopic lymphoid organs.^32^ A recent study has also shown that IL‐17 induces CXCL12 and iBALT development in response to *Pseudomonas aeruginosa* infection, where the main source of IL‐17 was *γδ* T cells (T*γδ*17 cells).^33^ The similarities in effector characteristics between Th17, LTi cells and *γδ* T cells may therefore account for the ability of these populations to drive ELF development (Fig. 1).

T follicular helper (Tfh) cells promote B‐cell activities and support the generation of high‐affinity antibodies at germinal centres.^34^, ^35^ Plasticity among effector T helper cells may also contribute to ELF development. For example, Th17 cells are linked with ELF development in the central nervous system, lungs and inflamed joint tissue.^18^, ^19^, ^21^ Interestingly, in the central nervous system Th17 cells develop a ‘Tfh‐like’ phenotype that may contribute to ELF development and function.^19^ ELF development during inflammatory arthritis is also linked with the local expression of Th17 and Tfh effector cytokines and transcription factors.^21^ Similarly, Th17 cells that home to Peyer's patches can acquire Tfh‐like effector characteristics that support antigen‐specific IgA responses at germinal centres.^36^ Here, Th17 cells recruited to the intestine express podoplanin. Therefore, lineage plasticity may provide the ability for effector T cells to develop Tfh‐like properties that support the development, maintenance and function of ELFs. Indeed, T helper cell plasticity is not solely confined to Th17 cells, and both Th1 and Th2 cells retain the ability to acquire the IL‐21, CXCR5, Bcl‐6, programmed cell death‐1 and inducible T‐cell co‐stimulator expression that are characteristic of Tfh cells (Fig. 1).^19^, ^36^, ^37^, ^38^ Therefore, other subsets beyond Th17 cells may soon emerge as initiators of ELFs.

Inflammatory cells may substitute for LTi cells in ectopic lymphoneogenesis, but there is increasing evidence that stromal tissue cells also display LTo‐like properties^39^, ^40^, ^41^. In rheumatoid arthritis, synovial fibroblasts contribute to ELF formation through the secretion of homeostatic chemokines such as CXCL13, CCL21 and CXCL12.^5^, ^23^, ^42^, ^43^, ^44^ Interestingly, synovial fibroblasts can also contribute to other aspect of ELF activity, where they can produce B‐cell‐activating factor and a proliferation‐inducing ligand (known as APRIL).^45^ These factors support activation‐induced cytidine deaminase (AID) expression, which drives somatic hypermutation and antibody class‐switching in B cells.^45^ Here, it is important to understand the relationship between the stromal tissue compartment and the nature of the inflammatory infiltrate because ELFs are not a universal feature of synovitis in inflammatory arthritis and only occur in a certain cohort of patients.

## Homeostatic chemokines in ELF development

The expression of homeostatic chemokines is increased in tissues where ELFs have emerged in response to foreign or auto antigens.^1^, ^9^, ^10^, ^12^ Chemokines such as CXCL13, CCL19, CCL21 and CXCL12 are involved not only in the initiation of ELF development, but also in the maintenance of the highly organized cellular architecture of established ELFs and SLOs. Given that the early clustering of LTi cells within the embryo is dependent on CXCL13, and that CXCL13 is detected early in the developing lymph nodes of LT*α*‐deficient mice,^46^, ^47^ this chemokine represents a key initiator of lymphoid organogenesis that functions upstream of LT*β*R signalling. At ELFs, CXCL13 and CCL21 regulate B‐cell and T‐cell infiltration and segregation at ELFs.^48^, ^49^, ^50^ Similarly, chemokines CCL19 and CXCL12 drive lymphocyte recruitment and the positioning of follicular DCs, B cells and plasma cells at germinal centres.^48^ Here, an elegant cooperation between CXCL12 and CXCL13 directs the movement of B cells from the dark zone into the light zone as they mature into antibody‐secreting plasma cells.^51^ Hence homeostatic and certain inflammatory chemokines contribute to both the initiation of ELF development, the cellular organization required for their function as germinal centres.

In addition to their chemotactic properties, homeostatic chemokines promote the secretion of LT*α*_1_ *β*_2_ by B cells and T cells, which establishes a feedback loop to perpetuate lymphocyte recruitment and positional organization.^49^, ^50^, ^52^ Interestingly mice lacking CXCL13, or its receptor CXCR5, fail to develop peripheral lymph nodes, underlining the importance of this chemokine in lymphoid organogenesis.^50^, ^52^, ^53^ Transgenic overexpression of *Cxcl13* in the pancreas induces the production of LT*α*_1_ *β*_2_ by B cells that is required for the development of ELFs that feature T‐cell and B‐cell segregation with HEV formation.^49^ CCL19 and CCL21 similarly drive the expression of LT*α*_1_ *β*_2_ on naive CD4 T helper cells.^48^ Hence, homeostatic chemokines are an integral feature of ELF development, organization and function. Nevertheless, there appears to be some hierarchy among these chemokines in their ability to drive ectopic lymphoneogenesis. For example, transgenic overexpression of *Ccl21* promotes the development of larger lymphoid follicles than those that emerge in response to *Ccl19*.^48^ Transgenic *Ccl21* expression also promotes the development of ELFs that display higher cellular organization than *Ccl19*. Interestingly, ectopic *Cxcl12* expression induces ELFs that contain few T cells but are enriched for follicular DCs, B cells and plasma cells.^48^ Therefore, as well as determining the size and degree of cellular organization within ELFs, the relative expression of homeostatic chemokines at inflammatory lesions will also determine the cellular composition of ELFs.

It is clear that some chronically inflamed tissues are more permissive to ELF development than others. For example, transgenic expression of *Ccl21* in the pancreas promotes ELFs that display T‐cell and B‐cell segregation, HEV and stromal reticulum. However, this response is context dependent and *Ccl21* in the skin fails to initiate lymphoneogenesis.^54^, ^55^ Histological features of ELFs are seen in various chronic inflammatory diseases and are clinically observed in the lung, joint synovium, liver, thymus, and salivary and thyroid glands (for a comprehensive review of ELFs in human disease refer to Pitzalis *et al*.^10^). Structurally mature ELFs are not, however, seen in skin conditions despite local expression of *Ccl21*, which suggests that the immune setting and the interaction of the stromal compartment with resident and infiltrating cells has to be conducive for ELF development to occur.^56^, ^57^ This may reflect differences in innate sensing responses or a differential stromal response to chemokines and cytokines necessary for the initiation, expansion and maintenance of tissue ELFs.

## High endothelial venules at ELFs

The function of SLOs depends on HEV that express peripheral node addressin and mucosal addressin cell adhesion molecule, which regulate the entry of naive T cells into the lymph node. Similarly, HEVs are also observed in ELFs that develop in response to autoimmune disease, allograft rejection and cancers.^41^, ^58^, ^59^, ^60^, ^61^, ^62^, ^63^, ^64^ However, HEVs also develop in tissues that do not feature ELFs,^65^, ^66^, ^67^ which infers that HEV neogenesis occurs before the recruitment of peripheral T and B cells and the emergence of ELFs. In peripheral lymph nodes, LT*β*R signalling in DCs and endothelial cells promotes HEV formation and maturation.^68^, ^69^ However, the mechanisms responsible for HEV development in inflamed tissues are less clear. In human breast cancer, HEVs correspond with a heightened expression of LT*β* by mature DCs.^66^ Here, high densities of HEVs are associated with a reduced frequency of FoxP3^+^ regulatory T (Treg) cells. Similar data have also been obtained in an experimental model of carcinogen‐induced fibrosarcoma, where Treg cell depletion caused reduced tumour growth and increased HEV formation.^65^ Indeed, HEVs in solid tumours are regarded as positive prognostic indicators and patients are often disease free for longer, show reduced evidence of tumour metastasis and display improved survival rates.^9^, ^65^, ^66^, ^67^, ^70^ In primary and metastatic tumours, HEVs are associated with increased infiltration of naive, central memory and activated T cells that display a Th1 effector phenotype.^66^, ^67^, ^70^ A high density of extra‐tumoral and intra‐tumoral Th1 cells correspond with improved survival,^71^ where it is proposed that their local priming, possibly within ELFs, promotes antigen‐specific tumour responses.^72^, ^73^ In murine models of melanoma and lung carcinoma, the development of lymph node‐like vasculature allows naive T‐cell entry into tumours that delay tumour outgrowth.^74^ Therefore modulation of HEV development or function through targeting LT*α*_1_ *β*_2_ to the tumour or by inhibiting Treg activity may provide opportunities for novel immunotherapies.

## ELFs as inductive sites for anti‐pathogen immune responses

Chronic bacterial and viral infections can trigger inflammation that results in the development of ELFs (comprehensively reviewed elsewhere^75^, ^76^). For example, *Helicobacter* spp. and *Mycobacterium tuberculosis* bacteria, as well as influenza and hepatitis C viruses, have been linked with ELF development in mice and humans.^77^, ^78^, ^79^, ^80^, ^81^, ^82^, ^83^, ^84^ Interestingly, ELF formation in response to infection features at mucosal sites including lung, gastric and salivary gland tissues.^78^, ^82^, ^85^ ELFs that develop in response to microbiota are also important for maintaining intestinal homeostasis.^86^ There is considerable evidence linking ELFs with anti‐microbial and viral immunity. For example mice lacking spleen, lymph nodes and Peyer's patches mount robust B‐cell and T‐cell responses to influenza virus at sites of iBALT formation.^82^ Notably, in the absence of peripheral lymphoid organs, these mice tolerate higher virus doses than wild‐type mice, suggesting that the anti‐viral response generated at iBALT is not only protective but may also be less pathogenic than those generated in the periphery. Mice were able to show effective primary and memory responses to viral challenge via ELFs, associated with the emergence of influenza‐specific CD8 T cells and anti‐influenza nucleoprotein‐specific antibodies.^81^, ^82^ Development of iBALT in response to infection was again linked to CXCL13 and CCL21 expression locally,^82^ but was also dependent on CD11c^hi^ DCs.^80^ Here, DCs were required for the maintenance of iBALT, and DC depletion resulted in the regression of the ELFs resulting in a reduction in germinal centre reactions, the number of class‐switched plasma cells and anti‐viral serum antibodies.

Similar ELF‐associated anti‐pathogen responses are also described for *M. tuberculosis* and murine cytomegalovirus infection in mice. In pulmonary *M. tuberculosis* infection, ELF development was associated with CXCL13, CCL21 and CCL19 expression and the recruitment of functional CXCR5^+^ T cells.^77^, ^79^ T cells expressing CXCR5 displayed Tfh‐ and Th1‐like effector characteristics and were important for host survival, *M. tuberculosis* clearance, T‐cell localization within ELFs, and lymphoid follicle formation.^79^ In murine cytomegalovirus infection, lymphoid follicles that form in the salivary glands participate as inductive sites in oral mucosal immunity.^85^ Development of these salivary follicles was accompanied by a local expression of homeostatic chemokines and molecular markers such as AID and I*μ*C*α* (the non‐excised rearranged DNA of IgA class‐switching), which regulate germinal centre activities including somatic hypermutation and class‐switch recombination.^85^

Development of ELFs is therefore an integral part of anti‐microbial and anti‐viral immunity. Although the regulation of these ELF‐driven responses can occur independent of SLOs, their presence in infected tissues supports adaptive immunity and the activities of SLOs. In humans, ELF involvement is often associated with persistent infections. Here, a failure to eradicate pathogens may lead to aberrant adaptive immune responses at ELFs that, when inappropriately controlled, drive the onset of tissue damage and chronic inflammation and transition to autoimmunity or cancer.

## ELFs as regulators of anti‐tumour immunity

Current cancer immunotherapies aim to enhance the adaptive immune response to tumour antigens to overcome the immunosuppressive microenvironment of the tumour.^87^ In this context, it is therefore surprising that ELFs arise in tumours at all. However, ELFs have been described in numerous cancers including colorectal,^60^, ^88^ rectal,^89^ breast,^67^, ^90^, ^91^ ovarian^67^, ^92^ and germ cell^93^ cancers, as well as melanoma,^62^, ^67^, ^94^ mucosal‐associated lymphoid tissue lymphoma^39^ and non‐small cell lung carcinoma^95^, ^96^ (NSCLC; see Dieu‐Nosjean *et al*. for a comprehensive review of ELFs in cancer^9^).

Structures resembling lymphoid follicles are reported at all stages of cancer, including primary tumours and metastases,^97^, ^98^ and in tumours removed after chemotherapy.^9^, ^96^ A number of studies have reported a correlation between the density of ELFs and the degree of T‐cell and B‐cell infiltration in tumours.^94^, ^95^, ^96^, ^97^ This is interesting, given that the frequency of T‐cells displaying Th1 and cytotoxic effector characteristics are linked with improved patient survival in cancer.^9^, ^71^ Indeed, ELFs in melanoma and NSCLC are associated with favourable patient outcomes and represent prognostic indicators of disease progression.^94^, ^95^ In lung tumour‐associated ELFs, a high proportion of mature DCs correlate with gene signatures linked with Th1 cell activation and improved long‐term patient survival.^97^ Similar observations have been reported for B‐cell densities in NSCLC, where follicular B cells correlate with long‐term survival both in early‐stage disease and in advanced disease treated with chemotherapy.^96^ Therefore, ELF development may facilitate the recruitment of naive T and B cells from the peripheral pool via HEVs and homeostatic chemokines. Once established, T‐cell and B‐cell priming within ELFs may provide a local source of antigen‐specific effector cells to drive anti‐tumour immunity.

Of course it is also possible the ELFs develop as a consequence of cancer‐associated inflammation, and are simply spectators of the inflammatory process rather than participants in tumour‐specific immune responses. However, in experimental mouse models, effective T‐cell priming at tumour‐associated ELFs have been demonstrated, that occur independently of SLOs.^72^, ^99^ Similarly in human NSCLC tumour‐associated ELFs, the density of germinal centre B cells correlates with the number of antibody‐secreting plasma cells specific for endogenous tumour‐associated antigens.^96^ A recent study has also shown that Treg cells in lung adenocarcinoma suppress anti‐tumour responses within tumour‐associated ELFs.^100^ These studies support a role for ELFs as sentinels for the propagation of adaptive immunity against cancer antigens. Immunotherapies that support the activities of ELFs therefore represent promising anti‐cancer treatments. These may include delivery of regulatory homeostatic chemokines and cytokines or interventions that block Treg involvement at ELFs.^65^, ^100^

## ELFs as perpetuators of inflammation‐driven pathology

Ectopic lymphoid follicles are associated with various autoimmune and chronic inflammatory diseases including rheumatoid arthritis,^11^, ^101^, ^102^ Sjögren syndrome,^103^ multiple sclerosis,^104^ experimental diabetes,^105^ atherosclerosis,^106^, ^107^ inflammatory bowel disease^108^, ^109^ and chronic obstructive pulmonary disease^110^, ^111^ (for a comprehensive review of ELFs in autoimmunity see refs 10, 76). In contrast to their roles in infection and cancer, ELFs in chronic inflammatory diseases are primarily associated with disease exacerbation.

Rheumatoid arthritis is an example of an autoimmune disease where ELFs impact the course of disease and the response to therapy. In rheumatoid arthritis, synovitis is classed into three distinct pathotypes based on cellular and molecular signatures of inflammation – termed pauci‐immune (or fibroblast‐rich disease), diffuse and follicular.^11^, ^101^ Synovial ELFs are a prominent feature of the follicular form of synovitis and are described in approximately 30–50% of patients with rheumatoid arthritis.^11^, ^43^, ^102^, ^112^, ^113^ Notably, ELFs are described at all stages of the disease and include patients with early active disease who have not received biological agents, patents with more progressive forms of rheumatoid arthritis, and those that have already received biological intervention.^43^, ^112^, ^113^ Although the mechanisms contributing the development of this form of pathology are largely unclear, ELFs in rheumatoid arthritis are associated with heightened synovial expression of CXCL13, CCL21, CCL19 and CXCL12, and cytokines LT*α*_1_ *β*_2_ and IL‐7.^21^, ^44^, ^114^, ^115^ Importantly, these structures correlate with disease severity and are associated with local T‐cell priming and autoantibody production.^43^, ^116^, ^117^, ^118^ Plasma cells in rheumatoid synovitis produce autoantibodies against citrullinated protein/peptides (ACPA/anti‐CCP).^116^ Using an elegant human rheumatoid arthritis–severe combined immunodeficient (HuRA–SCID) mouse chimera model, Humby *et al*. demonstrate that the synovial microenvironment determines the functional maintenance of ELFs and that control of this process is independent of further leukocyte infiltration.^116^ In this context, grafted synovial tissues containing ELFs continued to secrete ACPA and express AID.^116^ Although ACPA is a prognostic marker of rheumatoid arthritis in patients with early/undifferentiated disease, detection of serum ACPA is not indicative of ELF‐associated synovitis.^113^ The presence of synovial ELFs is however associated with severe synovitis and patients with this form of disease remain challenging to treat and display a poor response to anti‐tumour necrosis factor (anti‐TNF) therapy.^112^, ^119^ Here synovial IL‐7 receptor expression also predicts a negative response to anti‐TNF intervention.^112^ Although ELFs frequently correlate with the degree of synovitis and the infiltration of T cells and B cells,^21^, ^113^ it is still unclear whether their presence reflects increased clinical severity or more rapidly progressing forms of rheumatoid arthritis.^113^ Importantly, ELFs have been linked to an inferior clinical response to anti‐TNF therapy.^112^ However, in patients with ELFs that showed favourable responses to anti‐TNF treatment, regression of synovial ELFs correlated with improved clinical outcome.^112^ Further studies are therefore required to better define how ELFs relate to clinical severity and therapeutic response to biological agents in rheumatoid arthritis.

Ectopic lymphoid follicles often form in transplanted human tissues where chronic inflammation is associated with allograft rejection. Tissues derived from kidney transplantation demonstrate that ELFs contribute to germinal centre reactions, local B‐cell maturation and alloimmune responses (anti‐HLA antibody generation). Whereas these observations support a role for ELFs in chronic terminal rejection,^120^ two studies suggest that ELFs may actual elicit beneficial outcomes.^121^, ^122^ These findings in experimental models of kidney and cardiac allografts emphasize that ELF development promotes the recruitment of T cells and B cells displaying inhibitory or regulatory phenotypes.^121^, ^122^ Such activities would be predicted to suppress destructive alloimmune responses and promote graft tolerance.

Although lymphoid neogenesis is typically associated with exacerbation of chronic inflammatory processes, a recent study demonstrates a protective role for ELFs in atherosclerosis.^107^ ELFs develop in the aorta of aged *Apoe* ^*−/−*^ mice where they correlate with disease severity.^123^ These structures were typically located adjacent to atherosclerotic plaques where they control immune cell trafficking, antigen presentation and T‐cell priming and differentiation.^107^ Hence, aortic ELFs may play an important role in establishing local T‐cell immunity during ageing in atherosclerosis.

## Concluding remarks and future perspectives on the therapeutic targeting of ELFs

Based on the current literature it is envisaged that two immunotherapeutic strategies are required to target ELFs. In infection and cancer it would be desirable to bolster the development and activity of ELFs to support antigen‐specific responses. In contrast, the neutralization of ELFs in chronic inflammatory diseases and transplantation would limit tissue damage and rejection. However, to date the number of clinical studies targeting ELF‐associated pathology remains limited.

One potential strategy is to disrupt the spatial arrangement of T and B cells in ELFs. In chronic renal allograft rejection, B‐cell depletion with rituximab (anti‐CD20 monoclonal antibody) had a limited impact on the maintenance of ELFs and biological intervention promoted expression of the B‐cell survival factor, B‐cell‐activating factor.^124^ In rheumatoid arthritis, patients with ELFs show an inferior clinical response to anti‐TNF therapy, which typically targets macrophage and stromal cell responses.^112^ Other licensed biological therapies including IL‐6 or IL‐6 receptor‐specific monoclonal antibodies (e.g. tocilizumab), T‐cell activation antagonists (e.g. abatacept) and Janus kinase inhibitors (e.g. tofacitinib) are likely to target pathways linked with ELF activity. However, although effective in attenuating inflammatory processes that reflect their broader modes of action, their efficacy in controlling ELF activity are untested.^10^, ^125^ Given their prominent roles in lymphoid neogenesis, targeting LT*αβ* (e.g. baminercept and pateclizumab) may prove effective in blocking ELF activity. Although clinically untested for ELF‐associated inflammation, targeting LT*β* has shown promise in pre‐clinical experimental models of disease.^126^, ^127^, ^128^, ^129^ Similarly, CXCL13 blockade has shown some promise in pre‐clinical studies for the treatment of experimental inflammatory arthritis, diabetes and Sjögren syndrome.^130^, ^131^, ^132^ Finally, given the role of IL‐21 in regulating Tfh cells and germinal centre reactions, a monoclonal antibody against IL‐21 (NNC0114‐0006) is currently in clinical trials for rheumatoid arthritis and systemic lupus erythematosus. These trials follow encouraging evidence for amelioration of disease following blockade of IL‐21 signalling in experimental inflammatory arthritis, systemic lupus erythematosus and graft‐versus‐host disease.^133^, ^134^, ^135^

In cancer, promoting ELF activity may support the development of protective antigen‐specific responses. As such, targeting cytokines such as LT*αβ* to tumours may have therapeutic potential as demonstrated in experimental melanoma.^72^, ^99^ Recent studies also suggest that inhibition of Treg cell activities could promote endogenous anti‐tumour immune responses at ELFs.^65^, ^100^ In support of such approaches, a suppressive role for Treg cells in iBALT development has also recently been reported.^136^

In recent years, we have seen an increasing appreciation for the importance of ELFs as inducible sites within tissues for generating immune responses to infections, tumours, autoantigens and alloantigens. Here, experimental models of disease provide greater insight into the mechanisms that govern the development, function and maintenance of ELFs. These studies have led to the identification of novel immune cell subsets and stromal cell populations and new regulatory mechanisms that reflect potential therapeutic targets for immunomodulation of ELF activity. Significantly, molecular signatures of ELFs also have the potential to yield biomarkers capable of stratifying patients into defined classes of disease based on pathology. These are likely to facilitate clinical decisions regarding the most appropriate therapy for patients with ELF‐associated disease. Although experimental animal models have been the frontrunners in defining mechanistic aspects of ELF development, clinical studies in human conditions have validated many of these findings and shed light on the likely efficacy of current biologicals. Our capacity to stratify patients for the presence of ELFs in early disease is improving.^11^ The challenge is to understand when and how to target drugs against these important structures, and to adapt clinical trials in accordance with the activities of ELFs in cancer, infection, autoimmunity and transplantation.

## Disclosures

The authors declare no competing interests.
